# Pronostic de la grossesse chez les mineures mariées en cours de scolarisation à Niamey, République du Niger

**DOI:** 10.11604/pamj.2020.37.274.25834

**Published:** 2020-11-25

**Authors:** Hamidou Soumana Diaouga, Maimouna Chaibou Yacouba, Mahamane Mobarak Salifou Abdou, Madeleine Garba Rahamatou, Nafiou Idi, Madi Nayama

**Affiliations:** 1 Service de Gynécologie-Obstétrique, Maternité Issaka-Gazobi, Niamey, Niger,; 2 Service de Psychiatrie, Hôpital National de Niamey, Niamey, Niger,; 3 Service de Gynécologie-Obstétrique, Maternité du Centre Hospitalier Régional de Niamey, Niamey, Niger

**Keywords:** Grossesse, mineure, élève mariée, Niamey, Niger, Pregnancy, underage girls, married student, Niamey, Niger

## Abstract

**Introduction:**

l´objectif de cette étude était de déterminer la fréquence et d´évaluer le pronostic maternel et périnatal lors de la grossesse suivi de l´accouchement chez les élèves mineures mariées, dans la ville de Niamey. **Méthodes:** c´était une étude cas-témoin des gestantes et parturientes de janvier 2018 au 31 décembre 2018 à la Maternité Issaka Gazobi de Niamey. Les élèves mineures (<18 ans) ont été comparées aux élèves âgées de 18-27 ans. Les paramètres sociodémographiques maternels, la morbi-mortalité maternelle et périnatale ont été analysées. Les statistiques usuelles et la régression logistique ont été utilisées pour analyser les résultats. Le seuil de signification a été fixé à une valeur de P-value (p<0,05).

**Résultats:**

la fréquence d´accouchement chez les élèves mineures était de 3,06%. Les mineures étaient plus assidues au suivi prénatal (46,7% vs 41,9%). La césarienne (ORa=2 [1,0-3,0]), l´éclampsie (ORa=2 [1,0-4,4]), l´épisiotomie (ORa=2[1,2-1,8]) et la dépression néonatale (P<0,05 (10,6% vs 5,8%)) étaient plus élevés chez les mineures que chez les adultes. La mortalité périnatale était élevée dans les deux groupes.

**Conclusion:**

nos résultats sont proches de ceux décrits dans d´autres études européennes et africaines. Les différences retrouvées pour les risques obstétricaux et périnatals semblent être liées aux facteurs sociodémographiques entourant ces gestantes mineures. Ces derniers devraient être pris en compte dans toute démarche de prévention des complications des grossesses chez les mineures.

## Abstract (en)

**Introduction:**

the purpose of this study was to determine the frequency and to evaluate maternal and perinatal prognosis during pregnancy and childbirth among married child students in the city of Niamey.

**Methods:**

we conducted a case-control study of gestants and parturients at the Issaka Gazobi Maternity Hospital in Niamey over the period January 2018-December 31, 2018. Child students (<18 years) were compared to students aged 18-27 years. Maternal sociodemographic parameters and maternal and perinatal mortality were analyzed. The usual statistics and logistic regression were used to analyze the results. The significance level was set at p <0.05.

**Results:**

the frequency of delivery among child students was 3.06%. Minors attended prenatal follow-up (46.7% vs 41.9%) more diligently than adults. Cesarean section (ORa=2 [1.0-3.0]) eclampsia (ORa=2 [1.0-4.4]), episiotomy (ORa=2[1.2-1.8]) and neonatal depression (p <0.05 (10.6% vs 5.8%)) were higher among minors than among adults. Perinatal mortality was high in both groups.

**Conclusion:**

our results are close to those described in other European and African studies. The differences in obstetric and perinatal risks seem to be related to socio-demographic factors of child mothers. These should be taken into account in any approach to the prevention of pregnancy complications in minors.

## Introduction

Les jeunes filles âgées de 15 à 19 ans tombent enceinte chaque année dans les régions en développements dont 12 millions d´entre elles mettent au monde des enfants. Au moins 777,000 sont âgées de moins de 15 ans. Ceux sont l´Asie de l´Est et l´Afrique de l´Ouest qui comptent le plus grand nombre de naissance [1]. Au Niger l´Enquête Démographique et de Santé (EDSN) de 2017 a révélé que les adolescentes constituent 24,1% de l'ensemble des femmes en âge de procréer [2]. Elles contribuent pour près de 20% à la fécondité totale des femmes. Le taux de mariage des adolescentes est le plus élevé au monde; 75% des filles sont mariées avant 18 ans et 28% avant 15 ans [3]. L´âge légal du mariage est de 15 ans au Niger. Si la grossesse est un accomplissement dans bien des cas, elle peut aussi constituer un handicap majeur dans certaines situations, comme quand elle survient lors d´un cursus de scolarisation ou de formation. Au Niger ces grossesses surviennent principalement dans un contexte de mariage [4]. Depuis l´année 2004, certains auteurs ont rapporté que le mariage et le niveau d´instruction améliorait le pronostic de la grossesse chez les adolescentes [5]. La baisse de la mortalité périnatale résulte bien sûr de l´amélioration de l´instruction et des conditions de vie, mais aussi de progrès médicaux et des programmes de santé publique qui permettent aux femmes d´y accéder [6]. Peu d´études ont été rapportées sur l´impact du mariage et de la scolarisation sur le pronostic de la grossesse chez les mineures. Le but de cette étude était de déterminer la fréquence de l´accouchement chez les élèves mineurs mariées, de décrire leurs caractéristiques sociodémographiques et d´évaluer le pronostic maternel et périnatal lors de la grossesse suivi de l´accouchement chez les élèves mineurs mariées dans la ville de Niamey, à travers une première au Niger.

## Méthodes

Cinq cent cinquante-deux (552) élèves mariées qui se sont présentées à la maternité Issaka Gozobi pour un accouchement ou pour une complication gravido-puerpérale durant la période d´étude qui était du 1^er^ Janvier 2018 au 31 Décembre 2018 ont été incluses dans l'étude quel que soit le lieu de suivi de la grossesse. Les patientes réparties en deux groupes, en fonction de leur âge: un groupe comprenant les femmes âgées de moins de 18 ans et un second groupe réunissant les femmes âgées de 18 à 27 ans. Un entretien a permis de recueillir les caractéristiques sociodémographiques, la morbi-mortalité maternelle et périnatale de la patiente ainsi que les antécédents obstétricaux. Une fiche d´enquête individuelle avait été élaborée à cet effet et la recherche de données complémentaires a été réalisée dans le dossier obstétrical.

Caractéristiques sociodémographiques maternelles: l´âge maternel (femmes de moins de 18 ans: mineure et de 18 à 27: adulte), la parité, le niveau d´étude et le suivi de la grossesse (une grossesse était considérée comme non suivie si aucune consultation prénatale (CPN) n´avait eu lieu, mal suivie si le nombre de CPN était inférieur à 4; et bien suivie si ce nombre était supérieur ou égal à quatre.

Paramètres en rapport avec la morbi-mortalité maternelle: l´accouchement par césarienne, la présentation fœtale vicieuse (autre que le sommet), l´épisiotomie, l´anémie, la notion de transfusion, l´éclampsie, la délivrance pathologique, la lésion de parties molles, les complications maternelles, le décès maternel. Les lésions des parties molles inclus les déchirures cervicales, vaginales et périnéales. L'anémie a était défini par un taux d'hémoglobine inférieur à 11g/dl. Dans le paramètre «complications maternelles», nous avons regroupé toute complication maternelle notée en peripartum dont les infections, l´anémie, la RPM, l´éclampsie, l´hémorragie, la psychose, les thrombophlébites. L´état général était jugé sur la base de l´anémie, l´amaigrissement, l´asthénie. Il est bon en l´absence d´anémie sévère, d´amaigrissent et d´asthénie.

Paramètres en rapport avec la morbi-mortalité périnatale: le faible poids de naissance(FPN) (<2500 grammes), la dépression néonatale (score d´Apgar à la cinquième minute <7), la prématurité, la mort fœtale in utéro, le décès périnatal. Le test de khi2 d´indépendance (corrigé si nécessaire) ou du test exact de Fisher pour les variables qualitatives et le test de Student pour les variables quantitatives. L´odds ratio (OR) et ses intervalles de confiance à 95% (IC95%) ont été calculés. Le seuil de significativité était fixé à p<0,05. Les analyses ont été réalisées à l'aide des logiciels Epi Info 7.1 et Stata 12.

**Aspects éthiques et déontologiques:** une lettre officielle d´autorisation pour mener la recherche sur le terrain a été adressée au directeur général de la Maternité Issaka Gazobi de Niamey par la Faculté des Sciences de la Santé de l´Université de Niamey. La direction de la maternité a répondu favorablement pour mener l´enquête. Un consentement libre et éclairé de toutes les personnes impliquées dans cette étude a été obtenu verbalement.

## Résultats

**Fréquence:** sur un total de 5,807 accouchées enregistrées au cours de la période d'étude, nous avons répertorié 178 accouchées parmi les 184 mineures élèves mariées, soit une fréquence de 3,06%.

**Caractéristiques sociodémographiques, suivi de la grossesse et notion de référence de la population étudiée:** le Tableau 1 présente les caractéristiques sociodémographiques, le suivi de la grossesse et la notion de référence de la population étudiée. L´âge moyen était de 15,7 ans chez les mineures avec des extrêmes allant de 14 ans et 17 ans. La moyenne d´âge était de 20,4 ans dans le groupe témoin. Les Zarma-Sonraî constituaient l´ethnie la plus retrouvée dans le deux groupes (cas 53,8%, témoin: 44,8%). Les Kanouri et les Arabes étaient les seules ethnies épargnées par le phénomène du mariage et grossesse des élèves mineures dans notre étude. Quatre-vingt-trois virgule quinze pourcent (83,15%) des mineures fréquentaient une école publique contre 61,42% dans le groupe témoin (P<0,05). Treize virgule cinquante-huit pourcent (13,58%) des patientes mineures avaient doublé au moins deux années au cours de leur cursus scolaire contre 38,59% dans le groupe témoin (P<0,05). Plus de 2/3 (73,34 %) des mineures était au collège contre 32,87% dans le groupe témoin. Chez les mineures ce sont les classes de sixième et de seconde qui étaient les plus représentées avec respectivement 36,41% et 22,82%. Par contre chez les patientes de 18 ans ou plus, les classes de troisième et terminale ont été plus représentées avec respectivement 27,72% et 38,04% (P<0,05). L´âge moyen du mariage était de 15,9 ans contre 18,3 ans dans le groupe témoin (P<0,05). Seize mineures soit 8,7% étaient mariées avant 15 ans contre 0,5% dans le groupe témoin.

**Tableau 1:** caractéristiques sociodémographiques, le suivi de la grossesse et la notion de référence chez les mineures à Niamey

Paramètres	<18 ans (N=184)		18-27ans (N=368)		P
	**n**	**(%)**	**n**	**(%)**	
**Age maternel (ans)**					
Moyenne	15,7		20,4		
(Extrêmes)	14-17		18-27		
**Age du mariage (ans)**					
Moyenne	15,9		18,3		<0,001
(Extrêmes)	13-17		13-26		
**Intervalle mariage-grossesse (moi)**					
Moyenne	4±0,5		6±1,3		<0,001
(Extrêmes)	1-15		1-25		
**Parité**					
Moyenne	1,1		1,7		<0,001
(Extrêmes)	0-3		0-6		
0	1	0,5	4	1,1	<0,001
1	169	91,8	281	76,3	
2-3	14	7,7	77	21	
≥4	0	0	6	1,6	
**Etat civil**					
Célibataire	0	0	0	0	-
Mariée	184	100	368	100	
**Niveau d'étude**					
Primaire	2	1,08	0	0	<0,001
Collège	133	73,34	121	32,87	
Lycée	49	26,66	247	67,13	
**Profession du mari**					
Travailleur	176	95,66	361	98,1	<0,001
Etudiant/élève	8	4,34	7	1,9	
Sans emploi	0	0	0	0	
**Référence**					
Référée	134	72,8	265	72	0,836
Non référée	50	27,2	103	28	
**Nombre de CPN**					
Moyenne	3,9		3,01		0,104
(Extrêmes)	0-8		0-6		
0	8	4,4	11	3	0,104
1-3	90	48,9	203	55,1	
≥4	86	46,7	154	41,9	
**CPN**: consultation prénatale					

Les époux des patientes étaient des fonctionnaires dans 26,08% de cas contre 33,70% dans le groupe témoin (P=0,0004). Les primigestes ont été le plus représenté dans le deux groupes (cas: 91,3%; témoin: 73,6%) (P<0,05). La moyenne de la parité était de 1,01 chez les accouchées mineures contre 1,7 chez celles âgées de 18 ans ou plus avec une différence statistiquement significative (P<0,05). Dans les deux groupes aucune patiente ne fumait, ne buvait d´alcool avant et pendant la grossesse. La grossesse été survenue moins de quatre mois après le mariage chez 42,4% des patientes mineures et à moins d´un an du mariage chez 94,5% des cas. Ces taux étaient respectivement de 38,3% et 94% chez les élèves de 18 ans ou plus (P<0,05). La proportion des accouchées mineures référées par d´autres centres était supérieure à celle des témoins (72,8% vs 72%) mais nous n´avons pas observé une différence statistiquement significative (p=0,8366). Quarante-trois pourcent (43%) des mineures ont fait au moins une échographie obstétricale contre 46% dans le groupe témoin (P<0,05). La moyenne du nombre de CPN était de 3,9 avec une proportion de 4,4% des grossesses non suivies chez les mineures. Elle était de 3,01 avec une proportion de grossesses non suivies de 3% chez les patientes de 18 ans ou plus. Nous n´avons pas observé une différence statistiquement significative lorsque nous comparons les deux moyennes ainsi que les deux taux (p=0,1045). Cependant 46,7% des mineures avait réalisé au moins quatre CPN contre 41,9% dans le groupe témoin.

**Morbidité et mortalité maternelles:** le Tableau 2 présente pronostic maternel et périnatal chez la population d´étude. La rupture prématuré des membranes, la preéclampsie et l´éclampsie (ORa=2[1,0-4,4]) étaient les complications les plus retrouvées pendant la grossesse dans les deux groupes avec respectivement 27,2%, 20% et 21,4% chez les mineures contre 21,4%, 31,2% et 12,1% chez les élèves âgées de 18 ans ou plus. Chez sept virgule six pourcent (7,6%) des mineures, la présentation fœtale était vicieuse contre 4,3% chez les patientes âgées de 18 ans et plus; la différence observée n´était pas statistiquement significative (OR=1). La césarienne était le mode d´accouchement chez 54,5% des élèves mineures contre 46,2% dans le groupe témoin (P=0,0363, OR=1,7[1-2,3]). Les déchirures des parties molles ont été observées dans 23% des cas chez les mineures contre 26,4% chez les adultes (p=1). Chez les élèves mineures, la proportion d´épisiotomie était de 19,7% contre 12,2% chez celles âgées de 18 ans et plus. La dépendance était statistiquement significative (ORa=2[1,2-1,8]). La délivrance pathologique était plus retrouvée chez les mineures que chez les mères adultes. Cependant nous n´avons pas trouvé une dépendance statistiquement significative (P=1). Concernant la présence de complications maternelles en peripartum; elles étaient plus fréquente chez les mineures que chez les adultes (3,9% vs 1,3% (OR=2,9[1,3-4,5])).

**Tableau 2:** pronostic maternel et périnatal chez les élèves mineures

Paramètres	<18 ans (N=178)		18-27 ans (N=353)		OR brut [IC 95%]	OR ajusté [IC 95%]
	**N**	**%**	**n**	**%**		
Présentation vicieuse	13	7,6	15	4,3	1	-
Césarienne	97	54,5	163	46,2	1,7[1-2,3]	2[1,0-3,0]
Lésions des parties molles	41	23	93	26,4	1	-
Episiotomie	16	19,7	23	12,2	3,7[1,9-5,6]	2[1,2-1,8]
Délivrance pathologique	5	8,9	8	7,8	1	-
Présence de complications maternelles	7	3,9	5	1,3	2,9[1,3-4,5]	-
Eclampsie	15	8,5	15	4,3	3,4[1,8-5,7]	2[1,0-4,4]
Anémie en péripartum	85 (N=184)	46,3	127(N=368)	34,5	1	-
Transfusion	19 (N=184)	10,4	21(N=184)	5,8	1	-
Décès maternel	0	0	0	0	-	-
Score d'Apgar à la 5e minute<7	19	10,6	20	5,8	2[1,1-2,9]	1,8[1,0-3,1]
Naissance prématurée	33	18,4	82	22,6	1	-
PN<2500g	47	26,1	94	26,5	1,1[0,6-1,3]	1[0,8-1,1]
Décès périnatal	23	12,7	45	13,3	1	-

**g** : grammes ; **PN** : Poids de Naissance

L´éclampsie notée en peripartum était plus fréquente chez les mineures que chez les adultes avec des proportions respectives de 8,5% et 4,3%. Comparée à une mère adulte, une mère mineure présente plus de risque de faire l´éclampsie en peripartum (P<05, ORa=2[1,0-4,4]). L´anémie a été retrouve chez 46,3% de mineure dont 9,5% d´anémie sévère contre 34,5% dans le groupe témoin avec 5% d´anémie sévère. La différence n´était pas statistiquement significative entre ces deux groupes (p=0,963). En recherchant la notion de transfusion en peripartum, nous avons noté que les mineure ont été plus transfusées que les adultes (10,4% contre 5,8%) mais aucune différence statistique significative n´a été notée en comparant ces deux proportions (p=0,1611). Enfin, aucun décès maternels n´a été noté dans la population d´étude. Vingt-cinq virgule un pourcent (25,1%) des élèves mineures sont sortie de la maternité avec une contraception efficace de longue durée dont 6% du DIU du postpartum. Ces taux étaient respectivement de 13,3% et 2,5% chez les élèves âgées de 18 ans ou plus. La différence était statistiquement significative (P<0,05).

**Morbidité et mortalité périnatales:** les paramètres en rapport avec la morbidité et la mortalité périnatale sont présentés dans le Tableau 2. Dans les deux groupes étudiés, la proportion des faibles poids de naissance (FPN) était statistiquement la même. Le taux de FPN était de 26,1% chez les mineures contre 26,5% chez les élèves adultes (ORa=1[0,8-1,1]). La moyenne de poids de naissance était de 3700±536 grammes chez les nouveau-nés des mineures alors qu´elle était de 3750±550 grammes chez ceux nés des adultes. Nous n´avons pas observé une différence statistique significative entre ces deux moyennes (p=1). Dix-huit virgule quatre pourcent (18,4%) des nouveaux nés des mères mineures étaient prématurés contre 22,6% de ceux nés des mères adultes (p=1). La proportion de nouveau-nés déprimés à la 5^e^ minute de vie (score d'Apgar <7) était de 10,6% chez les mineures contre 5,8% dans le groupe des adultes. En comparant ces deux proportions, la différence était statistiquement significative (p<0,05). La dépression néonatale était deux fois plus fréquente chez les nouveau-nés des mères mineures (OR=1,8[1,0-3,1]). Une réanimation néonatale étaient réalisée chez 13,3% et 11,3% respectivement des nouveau-nés des mères mineures et adultes; la comparaison entre ces deux taux n´était pas statistiquement significative (p=0,104). S´agissant du décès périnatal, nous avons enregistré 12,7% de décès chez les enfants des mères mineures contre 13,3% chez ceux des mères adultes. La différence n´était pas statistiquement significative (P=0,2177). La grossesse a abouti à la naissance d´un enfant vivant en bonne santé dans 90,7% chez les mineures contre 86,7% dans le groupe témoin; mais nous n´avons pas trouvé une dépendance statistiquement significative (P=0,2177).

## Discussion

**Fréquence:** dans ce travail, nous rapportons les données d´une étude de 184 patientes élèves mineures mariées comparées à une population de 368 élèves plus âgées; prises en charge pour grossesse et accouchement en 2018 dans la maternité Issaka Gazobi de Niamey au Niger. Cette étude rapporte une fréquence de 3,06%. La fréquence de l´accouchement chez les mineures varie de 0,65% à 6,2% selon les auteurs [7,8]. En France, les départements et régions d´outre-mer restent les zones dans lesquelles les pourcentages de maternité chez les mineures sont les plus élevés avec des chiffres de 6,2% en Guyane, 3,8% à la Réunion [7]. Guiot O *et al*. [7] en Guadeloupe ont rapporté une fréquence de 4,2% des cas. Cette différence s´expliquerait par le fait que notre population d´étude est constituée uniquement des élèves mariées. Notre taux est supérieur à celui d´Alouini S *et al*. [8] en France qui ont noté 2,38% des cas. Notre taux (3,06%) s´expliquerait par le fait qu´au Niger à l´instar des pays en développement; les femmes sont soumises aux règles sociales et religieuses qui les obligent à se marier et fonder leur famille lorsqu´elles sont encore très jeunes. Les élèves n´échappent pas à ces contraintes. Le Niger fait partie des pays où le mariage d´enfant est le plus pratiqué au monde [9-11].

De nombreuses adolescentes subissent le choix de leurs parents et la pression sociale. Elles sont subitement projetées dans le monde des adultes avec des responsabilités de femme et de mère. Le mariage est également perçu comme un moyen d´éviter les grossesses précoces hors mariage. La fécondité conditionne, dans une certaine mesure, le statut de la femme dans la société. Quelle que puisse être la volonté de la femme de chercher une promotion sociale à travers l´école, elle est toujours rattrapée par son rôle attendu de mère [1,10]. Cette fréquence pourrait aussi s´expliquer par le fait qu´au Niger; la population est en majorité très jeune, 48,8% de la population est âgée de 15-19 ans. L´indice synthétique de fécondité moyen est l´un des plus élevés du monde avec un taux de 6 enfants par femme, cet indice est de 5,3 enfants par femme à Niamey [2].

**Caractéristiques sociodémographiques et suivi prénatale:** les accouchées mineures avaient un âge moyen de 15,7 ans et une parité moyenne de 1,01. Cette valeur est supérieure à la moyenne nationale de la première naissance au Niger qui est de 18,1 ans [12]. Mais cette dernière concerne toute les filles en cours de scolarisation sans distinction d´âge et de statut matrimonial. La forte prévalence de la grossesse et de la maternité précoces était observée dans plusieurs études Africaines et Européennes. Ainsi, Guiot *et al*. [7] en Guadeloupe ont rapporté un âge moyen de 16,01 ans. Ndayizeye J *et al*. [13] ont rapporté un intervalle d´âge entre 14 et 22 ans dont plus de la moitié était âgée de 14 à 18 ans. Gbaguidi T [14] notifiait que 54,19% des gestantes en cours de scolarisation avaient un âge compris entre 15 et 17 ans, Ka A *et al*. [15] ont rapporté 69% de cas de la tranche d´âge entre 16 et 19 ans. Goguoa R [16] a notifié 40% des cas d´élèves enceintes entre 15 et 18 ans. L´âge moyen du mariage était de 15,9 ans chez les mineures contre 18,3 ans chez les adultes. Ce taux est similaire à l´âge moyen national du premier mariage au Niger qui est de 15,7 ans [2].

Ceci s´expliquerait par les politiques de maintien des filles à l´école mise en place par l´Etat du Niger avec l´appui des partenaires, pour retarder l´âge du mariage chez les filles en cours de scolarisation. Aucune mineure n´avait une contraception avant la survenue de la grossesse; contre 1,6% pour les adultes. Le caractère tabou de la sexualité au Niger rend les mineures réticente à recourir aux services de santé de la reproduction. Concernant le suivi prénatal, nos résultats ne montrent pas une différence significative lorsque nous comparons les moyennes de nombre de CPN (3,9 vs 3,01). Mais cette différence était significative en ce qui concerne les taux des grossesses bien suivies (46,7% vs 41,9% (P<0,05)). Cinquante-trois virgule trois pourcent (53,3%) des accouchées mineures n´ont pas bénéficié de CPN bien suivies contre 58,3% pour les élèves adultes. Le rôle de la CPN bien conduite est essentiel. Cela permettrait une réduction significative des différentes complications survenant au moment de la grossesse et de l´accouchement. Le suivi prénatal est la période privilégiée où les grossesses à risque sont décelées en vue d´une prise en charge [6,7].

**Morbidité et mortalité maternelles:** l´étude montre que la présentation fœtale vicieuse, les lésions des parties molles, l´anémie et la notion de transfusion n´ont pas été statistiquement liés au jeune âge (p>0,05). Cependant, les accouchées mineures avaient statistiquement un risque plus élevé d´accoucher par césarienne (ORa=2), de subir une épisiotomie (ORa=2), d´avoir une délivrance pathologique (8,9% vs 7,8%), de présenter l´éclampsie en peripartum (ORa=2) et de manière générale, de complications maternelles gravido-puerpérale (ORa=2,9). La césarienne était significativement plus fréquente chez les mineures que chez les patientes âgées de 18-27 ans (ORa=2). Cette tendance a été retrouvée dans d´autres études [7,17]. Les bassins des adolescentes, avant 16 ans, sont souvent de dimensions insuffisantes et correspondent à des bassins généralement rétrécis. Ce qui est responsable des complications obstétricales fréquentes, principalement en dessous de 15 ans [17]. Après 18 ans, on peut considérer les dimensions pelviennes comme définitives [18]. Le taux d´éclampsie est inversement proportionnel à l´âge de la femme dans notre série. La survenue de l'éclampsie était plus fréquente chez les moins de 18 ans (ORa=2,1). Cette association entre l´éclampsie et le jeune âge a été aussi retrouvée dans d´autres études [7]. Plusieurs auteurs ont rapporté une fréquence élevée de l´hypertension artérielle au cours de la grossesse chez la femme très jeune et ont incriminé l´immaturité biologique et endocrinienne, la primigestité et le manque de suivi prénatal [17].

L´épisiotomie était pratiquée environ 2 fois plus chez les mineures que chez les adultes (ORa=2). Ce constat est retrouvé dans d´autres études [17]. L´immaturité du périnée pourrait expliquer ce taux élevé d'épisiotomie. En recherchant la notion de transfusion et de l´anémie en peripartum, aucune différence significative entre les deux groupes n´a été notée (P>0,05).Mais le taux d´anémie était plus élevé chez les mineures que chez les adultes (46,3% vs 34,5%). Nos résultats sont comparables à ceux de Luhete PK *et al*. [17] qui trouvaient un risque élevé de développer une anémie au décours d´un accouchement chez des adolescentes. S´agissant de la mortalité maternelle, nous n´avons enregistré aucun décès maternel dans les deux groupes. D´autre études africaines et européennes ont rapporté un taux de décès maternel statistiquement indépendant de l´âge (p>0,05) [7,17]. Dans notre série la grossesse a abouti à la naissance d´un enfant vivant dans 90,7% de cas (contre 86,7% chez les adultes). Ce taux est largement supérieur à celui de Dagnogo GB [19] en Côte d´ivoire qui a rapporté 72,42%, et à celui de Gbaguidi T [14] au Bénin avec 44,52% des cas. Cette différence s´expliquerait par le fait que dans notre étude toutes les filles étaient mariées. Aussi l´avortement demeure interdit au Niger.

**Morbidité et mortalité périnatales:** concernant les paramètres de naissance des nouveaux nés; les mères mineures présentaient un risque similaire (P>0,05) d´accoucher un FPN mais la prématurité était plus fréquente chez le nouveaux nés de mère adulte (cas: 18,4% témoins: 22,6%). Nos résultats sont en contradiction avec d´autres études. Ainsi certains auteurs ont rapporté un taux plus élevé de prématurité et d´hypotrophie chez les nouveaux nés de mère mineure [7,20,21]. Cependant dans notre étude les mineures étaient plus assidue aux suivis prénatals et ont un niveau socio-économiques similaires aux à celui des adultes. Le mariage, le niveau d´instruction, la qualité du suivi prénatal et d´accouchement médicalement assisté constituent des facteurs de bon pronostic de la grossesse chez ces mineures [5]. L´étude montre une proportion élevé des nouveau-nés déprimés à la 5^e^ minute de vie (score d'Apgar <7) chez les mineures par rapport à ceux des adultes (10,6% contre 5,8%). L´analyse donne une différence statistiquement significative (ORa=1,8; P<0,05). La mortalité périnatale était moins élevée chez les nouveau-nés des mères mineures que chez ceux des mères adultes (12,7% vs 13,3% (OR=1)). Notre résultat est en contradiction avec certains auteurs [7]. Cependant, il existe d´autres études qui n'ont pas trouvé une association entre le jeune âge maternel et la mortalité périnatale [22,23]. C´est le moment de rappeler le rôle déterminant du niveau socio-économique, du plateau technique et de la qualité du suivi prénatal et l´accouchement assisté comme nous montre l´étude de Wemaux-Denis C *et al*. [24] en France.

## Conclusion

Cette étude montre qu´à niveau de prise en charge égal, la grossesse et l´accouchement chez les élèves mineures mariées présentent un pronostic similaire à celui de leurs homologues plus âgés. Nos résultats corroborent ceux d´autres études africaines, asiatiques et européennes. Cependant des efforts restent à fournir pour réduire la mortalité périnatale élevée chez les patientes mineures et leurs homologues adultes. L´accent doit être mis sur la contraception efficace et surtout, en cas de grossesse, sur l´amélioration de la qualité du soin prénatal, peripartum et postnatal. La sensibilisation pour une meilleure fréquentation des services des soins prénataux.

### Etat des connaissances sur le sujet

- Le phénomène du mariage et la grossesse des mineures en cours de scolarisation est un problème majeur de santé publique en république du Niger;
- Le pronostic de la grossesse chez les mineures dépend surtout du niveau socio-économique, de la qualité du suivi prénatal et des possibilités d´accouchement assisté par un personnel compétant; plutôt qu´au jeune âge;
- Le mariage et le niveau d´instruction constituent des facteurs de bon pronostic dans l´évolution de la grossesse chez les mineures.

### Contribution de notre étude à la connaissance

- Aucune étude sur ce sujet n´a déjà été publiée antérieurement sur les facteurs de risque et le pronostic maternel et périnatal de la grossesse suivi de l´accouchement chez les mineures en cours de scolarisation dans notre contexte au Niger;
- Notre étude apporte une analyse multivariée permettant d´exposer les données de la ville de Niamey (Niger) sur ce sujet;
- Notre étude montre que le pronostic de la grossesse chez les mineures serait meilleur en mettant l´accent sur le niveau socio-économique, la qualité du suivi prénatal et des possibilités d´accouchement assisté par un personnel compétant.
